# Inhibition of dynamin-dependent endocytosis increases shedding of the amyloid precursor protein ectodomain and reduces generation of amyloid β protein

**DOI:** 10.1186/1471-2121-6-30

**Published:** 2005-08-11

**Authors:** Robyn M Carey, Brigitte A Balcz, Ignacio Lopez-Coviella, Barbara E Slack

**Affiliations:** 1 Department of Pathology and Laboratory Medicine, Boston University School of Medicine, 715 Albany Street, Rm. L808, Boston MA 02118, USA; 2 Gemeinnützige Salzburger Landeskliniken Betriebsgesellschaft mbH, Universitätsklinik für Innere Medizin III, Paracelsus Medizinische Privatuniversität, Müllner Hauptstrasse 48, A-5020 Salzburg, Austria

## Abstract

**Background:**

The amyloid precursor protein (APP) is transported via the secretory pathway to the cell surface, where it may be cleaved within its ectodomain by α-secretase, or internalized within clathrin-coated vesicles. An alternative proteolytic pathway occurs within the endocytic compartment, where the sequential action of β- and γ-secretases generates the amyloid β protein (Aβ). In this study, we investigated the effects of modulators of endocytosis on APP processing.

**Results:**

Human embryonic kidney cells were transfected with a dominant negative mutant of dynamin I, an important mediator of clathrin-dependent endocytosis, and APP proteolysis was analyzed. Overexpression of the mutant dynamin (dyn I K44A) resulted in increased shedding of the APP ectodomain (sAPPα), accumulation of the C-terminal α-secretase product C83, and a reduction in the release of Aβ. Levels of mature APP on the cell surface were increased in cells expressing dyn I K44A, and internalization of surface-immunolabeled APP, assessed by fluorescence microscopy, was inhibited. Dynamin is a substrate for protein kinase C (PKC), and it was hypothesized that activators of PKC, which are known to stimulate α-secretase-mediated cleavage of APP, might exert their effects by inhibiting dynamin-dependent endocytosis. However, the internalization of surface-biotinylated APP was unaffected by treatment of cells with phorbol 12-myristate 13-acetate in the presence of the α-secretase inhibitor TAPI-1.

**Conclusion:**

The results indicate that APP is internalized by a dynamin-dependent process, and suggest that alterations in the activity of proteins that mediate endocytosis might lead to significant changes in Aβ production.

## Background

The amyloid precursor protein (APP) is a single-pass transmembrane protein that gives rise to the small peptides (known as Aβ) that form amyloid deposits in the brains of patients with Alzheimer's disease (AD) [1,2]. Aβ peptides are generated by the successive cleavage of APP by proteases known respectively as β- and γ-secretases. Alternatively, APP may be cleaved within the Aβ domain by α-secretases, now believed to be members of the disintegrin and metalloprotease (ADAM) family [3-5]. This latter process precludes the formation of Aβ, and results in the shedding of a large soluble N-terminal fragment of APP (sAPPα) into the extracellular or intra-luminal space. Cleavage of APP by α-secretases may occur in a late compartment of the secretory pathway, or at the cell surface [6].

APP ectodomain shedding occurs in both a constitutive and a regulated fashion. A key mediator of regulated shedding is protein kinase C (PKC), whether it is stimulated directly by phorbol esters, or as a consequence of the activation of receptors coupled to phosphoinositide turnover. Although the stimulation of APP shedding by PKC activators has been extensively documented [7], the mechanism is still unclear. Direct phosphorylation of the APP intracellular domain is not required, since phorbol esters are still able to increase shedding of a C-terminally truncated form of APP, or of APP constructs in which serine or threonine residues in the cytoplasmic domain have been replaced with alanine [8-10]. Likewise, C-terminal truncation of the putative α-secretase ADAM17/TACE (tumor necrosis factor-α converting enzyme) did not prevent up-regulation of its activity toward its substrate tumor necrosis factor-α (TNFα) by phorbol 12-myristate 13-acetate (PMA) [11]. On the other hand, phorbol ester-regulated cleavage of TrkA by TACE was found to be dependent, in part, on phosphorylation of threonine 735 within the TACE cytoplasmic domain [12]. Thus, phosphorylation of ADAM proteases may modulate their activity, at least toward certain substrates.

PKC-mediated effects on vesicular trafficking might also affect APP processing. A study showing that PKC activation increases the formation of APP-containing secretory vesicles from the trans-Golgi network [13], suggested that accelerated trafficking of APP to the cell surface might underlie the increase in sAPPα release induced by PKC. Alternatively, inhibition of endocytosis could increase sAPPα release by prolonging the interaction of APP with secretases on the cell surface. APP is found within clathrin-coated vesicles [14,15], which mediate the internalization of many cell surface proteins. Clathrin-dependent endocytosis is regulated by the high-molecular weight GTPase dynamin, which forms oligomeric rings around the neck of the forming vesicle, and severs it from the plasma membrane [16]. Dynamin activity, in turn, is reportedly governed by PKC [17-19], raising the possibility that PKC might modulate internalization, and therefore secretory cleavage, of APP, via an effect on endocytosis.

The aims of the present study were two-fold: to examine the effects of an inhibitor of dynamin function on APP processing, and to determine if PKC activation stimulates APP shedding via inhibition of endocytosis. Overexpression of a dominant negative dynamin mutant in HEK cells co-transfected with APP_695 _increased surface expression of APP and release of sAPPα, while inhibiting the internalization of full-length APP. The dynamin mutant also increased formation of C83, the C-terminal stub generated by α-secretase-mediated cleavage of APP, and reduced the release of Aβ peptides. These results contrast with a recent study, in which induction of dominant negative dynamin (dyn I K44A) increased both sAPPα release and Aβ formation [20]. Although activation of PKC by treatment with the phorbol ester PMA stimulates shedding of the APP ectodomain, PMA had no effect on the internalization of surface-biotinylated APP. Our observations provide direct evidence that APP internalization is a dynamin-dependent process. Moreover, the results indicate that activators of PKC do not promote sAPPα release via inhibition of endocytosis.

## Results

### Ectodomain shedding of APP is increased in cells transfected with dyn I K44A

**Figure 1:**
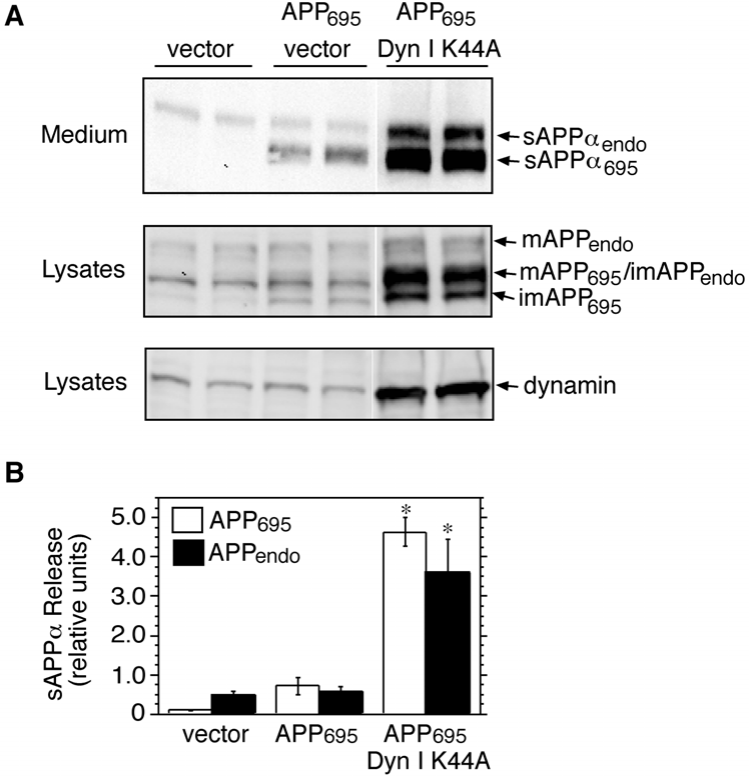
**Release of sAPPα is increased by dyn I K44A overexpression. **HEK-M3 cells were transiently transfected with APP_695 _and dyn I K44A or empty vector. After 48 hours, the growth medium was replaced with serum-free medium, and collected after 2 hours. Proteins in media extracts and cell lysates were size-fractionated on SDS gels and analyzed by immunoblotting. A. Expression of dyn I K44A increased release of endogenous and co-transfected sAPPα (upper panel; bands were detected with 6E10 antibodies) and levels of cellular APP (middle panel; bands were detected with anti-APP-CT). Arrows indicate mature (m) and immature (im) isoforms of endogenous and transfected APP. The mutant dynamin was expressed at high levels in transfected cells (lower panel). The lanes depicted in each panel were derived from the same blot. B. Levels of sAPPα in the media extracts were quantitated by densitometry and values were expressed as means ± SEM from 3 experiments. *, significantly different from the other two groups, by analysis of variance and Fisher's Least Significant Difference test.

The GTPase dynamin is an important mediator of clathrin-dependent endocytosis and synaptic vesicle recycling, and is required for the internalization of many cell surface proteins, including growth factor and G-protein coupled receptors [21]. To determine if dynamin regulates the internalization of APP, HEK-M3 cells were transiently transfected with APP_695 _and either an empty vector or a plasmid encoding the dominant-negative dynamin mutant dyn I K44A, which is deficient in GTP binding and GTPase activity [22]. The APP_695 _isoform was used for these transfection studies, since it is not expressed by HEK cells; and can be distinguished on western blots from the longer endogenous APP isoforms. Cells transfected with empty vectors alone were used as additional controls. Levels of sAPPα in the medium, and cellular full-length APP, were detected by western blotting using 6E10 antibodies, and antibodies to the APP C-terminus (APP-CT), respectively. The expression of endogenous dynamin, assessed using an antibody that recognizes both dynamin I and dynamin II isoforms, was low in cells transfected with APP_695 _or empty vector, and robust overexpression of the mutant protein was observed in cells transfected with the plasmid encoding dyn I K44A (Fig. 1A, lower panel). Dynamin I is neuron-specific, whereas dynamin II is widely expressed. The immunoreactive band present in cells that were not transfected with the dynamin plasmid presumably represents dynamin II, since no signal was detectable when lysates from these cells were immunoblotted with antibodies specific for dynamin I (not shown). An increase of approximately five-fold in the release of endogenous and transfected sAPPα from cells transfected with dyn I K44A was observed (Fig. 1A, upper panel, and Fig. 1B). Levels of cellular full-length APP were also increased in cells expressing the dynamin mutant (Fig. 1A, middle panel). Quantitation of the band comprising mature cellular APP_695 _and immature endogenous APP (APP_endo_) showed that levels in the presence of the mutant dynamin increased to 2.86 ± 0.97-fold control levels (mean ± SEM, n = 3). Overnight treatment of APP_695 _transfectants with the lysosomal protease inhibitor chloroquine (50 μM) increased levels of this band to a similar extent (to 2.30 ± 0.02-fold control, mean ± SEM from 3 experiments).

### Inhibition of dynamin function increases surface expression of APP

**Figure 2:**
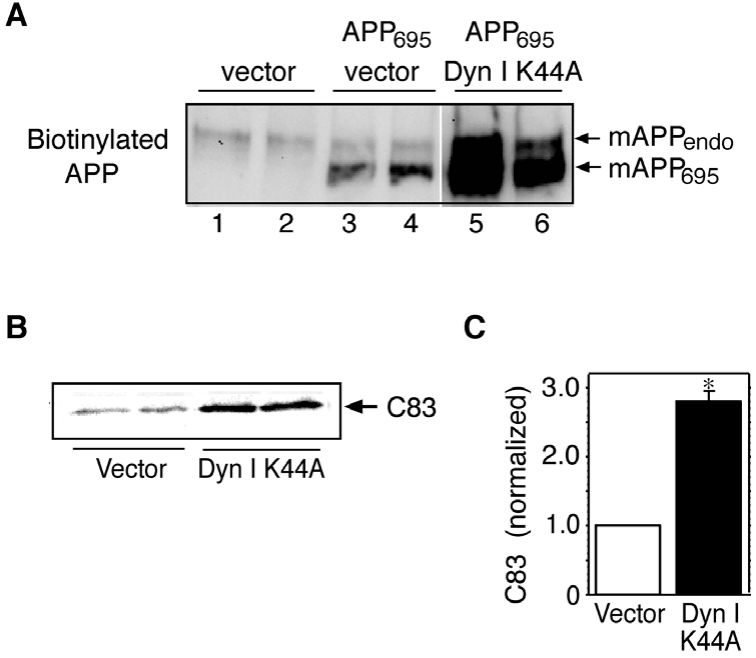
**Surface expression of APP is increased by dyn I K44A overexpression. **A. HEK-M3 cells were transiently transfected with APP_695 _and dyn I K44A or empty vector. After 48 hours, cells were surface biotinylated, lysed, and incubated with streptavidin-agarose beads. Biotinylated proteins were immunoblotted with antibodies to the APP C-terminal. Full-length, biotinylated, endogenous and co-transfected APP are indicated by arrows. B. HEK-695 cells were transiently transfected with dyn I K44A or empty vector. Immunoblot analysis of cell lysates showed increased formation of the APP C-terminal fragment (C83) generated by α-secretase cleavage in cells transfected with the dynamin mutant. C. Results from B were quantitated, normalized and expressed as means ± SEM from 3 experiments. *, p < 0.05 by paired t-test.

The effect of the dynamin mutant on surface expression of APP was next examined by surface biotinylation of transiently transfected HEK-M3 cells. In cells transfected with empty vector alone, one diffuse band of biotinylated APP representing mature endogenous APP was visible on western blots (Fig. 2A, lanes 1 and 2). Cells transfected with APP_695 _expressed an additional, more rapidly migrating band, representing mature cell surface APP_695 _(Fig. 2A, lanes 3 and 4). Surface expression of both endogenous and transfected APP_695 _was greatly increased in cells overexpressing dyn I K44A (Fig. 2A, lanes 5 and 6). As shown in Fig. 1, both mature isoforms of APP were released into the medium of APP_695 _transfectants, and release of both isoforms was markedly increased in cells overexpressing dyn I K44A.

### Expression of dyn I K44A increases formation of the APP C-terminal fragment C83

Cleavage of APP by α-secretase results in the release of the soluble ectodomain fragment sAPPα, and leaves a C-terminal stub, known as C83, in the cell membrane. In HEK cells stably overexpressing APP_695 _(HEK-695 cells) a protein corresponding in size to C83 was detected by western blotting of cell lysates with antibodies to the APP C-terminus (Fig. 2B). The corresponding β-secretase product C99 was not detectable under these conditions. Levels of C83 were significantly increased in cells transfected with dyn I K44A, relative to levels in cells transfected with empty vector alone (Fig. 2B and 2C), consistent with the increase in ectodomain shedding observed in cells expressing the dynamin mutant.

### Internalization of APP is inhibited in cells transfected with dyn I K44A

**Figure 3:**
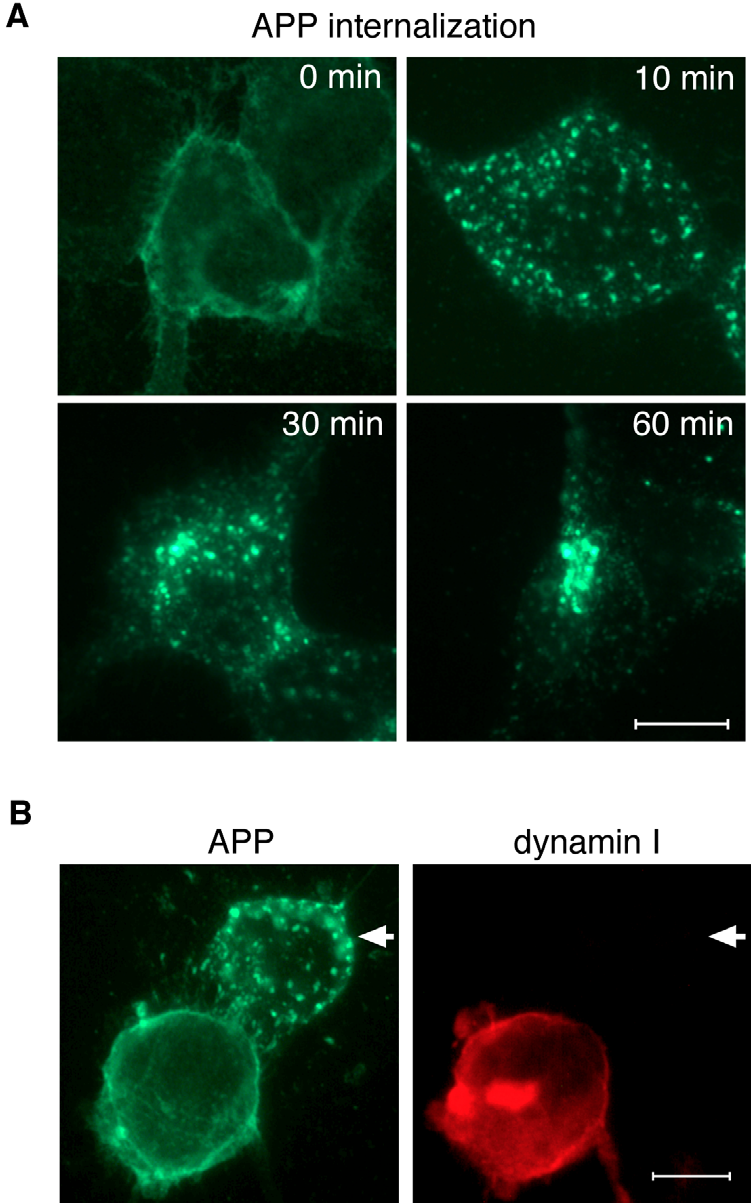
**APP internalization is inhibited in cells transfected with dyn I K44A. **A. HEK-695 cells were surface-immunolabeled with 6E10 antibodies for 45 min while on ice. The cells were then incubated at 37°C for varying periods of time, to allow internalization to occur. Prior to warming (0 min), APP immunofluorescence was confined to the cell membrane. Within 5 minutes APP immunoreactivity was located within punctate structures near the cell membrane. By 60 minutes, most of the immunoreactivity had coalesced at a perinuclear site. B. HEK-695 cells were transiently transfected with dyn I K44A. After 48 hours, cells were surface-labeled with 6E10 antibodies, then incubated at 37° for 10 min. In untransfected cells (arrows) APP (green) was internalized within intracellular vesicles within 10 minutes. In dyn I K44A-transfected cells (red), APP immunoreactivity was still largely membrane-associated at this time-point, indicating that internalization was impaired in cells expressing the dynamin mutant. Bar, 10 μm.

Our results can be interpreted to suggest that overexpression of dyn I K44A inhibits endocytosis of APP. The increase in cellular levels of APP observed in cells expressing the mutant dynamin, accordingly, could be the result of decreased internalization and degradation of full-length APP (Fig. 1A), and indeed, the lysosomal protease inhibitor chloroquine exerted a similar effect. However, it could also be argued that the increases in APP surface expression and shedding caused by the dynamin mutant were secondary to elevations in APP expression. Therefore, in order to directly examine the effect of dynamin on APP endocytosis, APP internalization in living cells was assessed using an immuno-labeling assay. Live HEK cells stably overexpressing APP_695 _were incubated at 4°C with 6E10 antibodies, in order to label cell surface APP. The cells were washed and warmed to 37°C for various intervals, and then fixed, permeabilized, and stained with Alexa 488-conjugated secondary antibodies. In cells that were labeled on ice and then fixed and stained prior to warming, APP was largely confined to the plasma membrane (Fig. 3A, 0 min). After 10 minutes at 37°C, most of the immunofluorescence was contained within intracellular punctuate structures distributed throughout the cytoplasm, indicating that surface APP had moved into an endosomal compartment. By 60 minutes, the immunofluorescent signal representing internalized APP had for the most part coalesced at a perinuclear site (Fig. 3A). Only background fluorescence was observed in cells that were incubated with antibodies to paxillin, an intracellular protein (not shown). APP internalization was next determined in these cells 48 hours after the cultures were transiently transfected with dyn I K44A. The cells were surface-labeled with 6E10 antibodies, and incubated at 37°C for 10 minutes. They were then fixed, permeabilized, and stained with Alexa 488-conjugated anti-mouse IgG to detect APP. Dynamin I expression was assessed by co-staining cells with goat anti-dynamin I antibodies followed by Alexa 594-conjugated anti-goat IgG. Immunoblot analysis of cell lysates confirmed that this antibody, which is specific for dynamin I, does not detect endogenous dynamin in non-transfected cells (not shown). In cells expressing the mutant dynamin I (Fig. 3B, right panel), APP immunoreactivity was restricted to the cell surface (Fig. 3B, left panel), whereas untransfected cells within the same culture exhibited a punctate pattern of APP immunofluorescence indicative of internalization (Fig. 3B, arrows). These results provide direct evidence that dyn I K44A inhibits endocytosis of APP.

### Overexpression of dyn I K44A inhibits the formation of Aβ peptides

**Figure 4:**
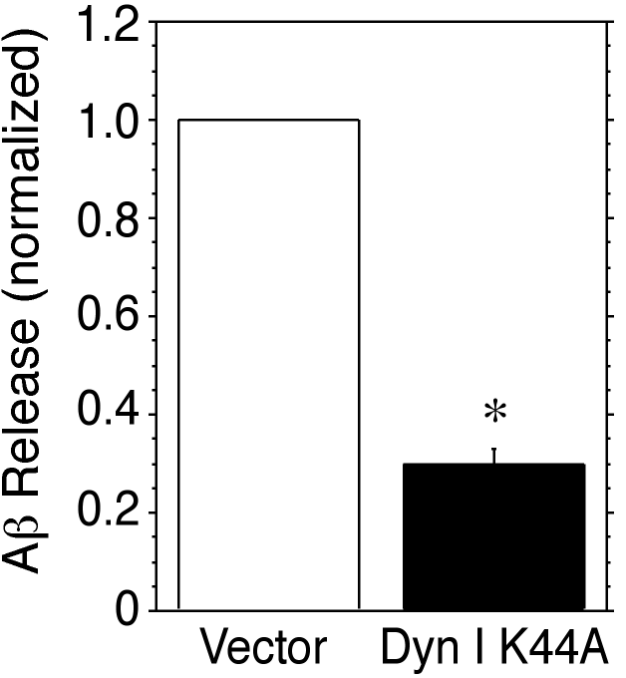
**Dyn I K44A inhibits Aβ formation. **HEK-695 cells were transiently transfected with empty vector or dyn I K44A. Medium was collected for 24 hours and analyzed for Aβ_1–40 _levels by sandwich ELISA. Levels of Aβ_1–40 _were significantly lower in the medium of cells transfected with dyn I K44A. *, p < 0.05 by paired t-test.

In order to determine if dynamin inhibition affects the formation of Aβ peptides, levels of Aβ_1–40 _in the medium of HEK-695 cells transiently transfected with either an empty vector or dyn I K44A were measured by ELISA. Overexpression of the dynamin I mutant caused a significant reduction in Aβ release (Fig. 4), suggesting that APP internalization is necessary for generation of Aβ in HEK cells.

### Activation of PKC does not affect internalization of APP

**Figure 5:**
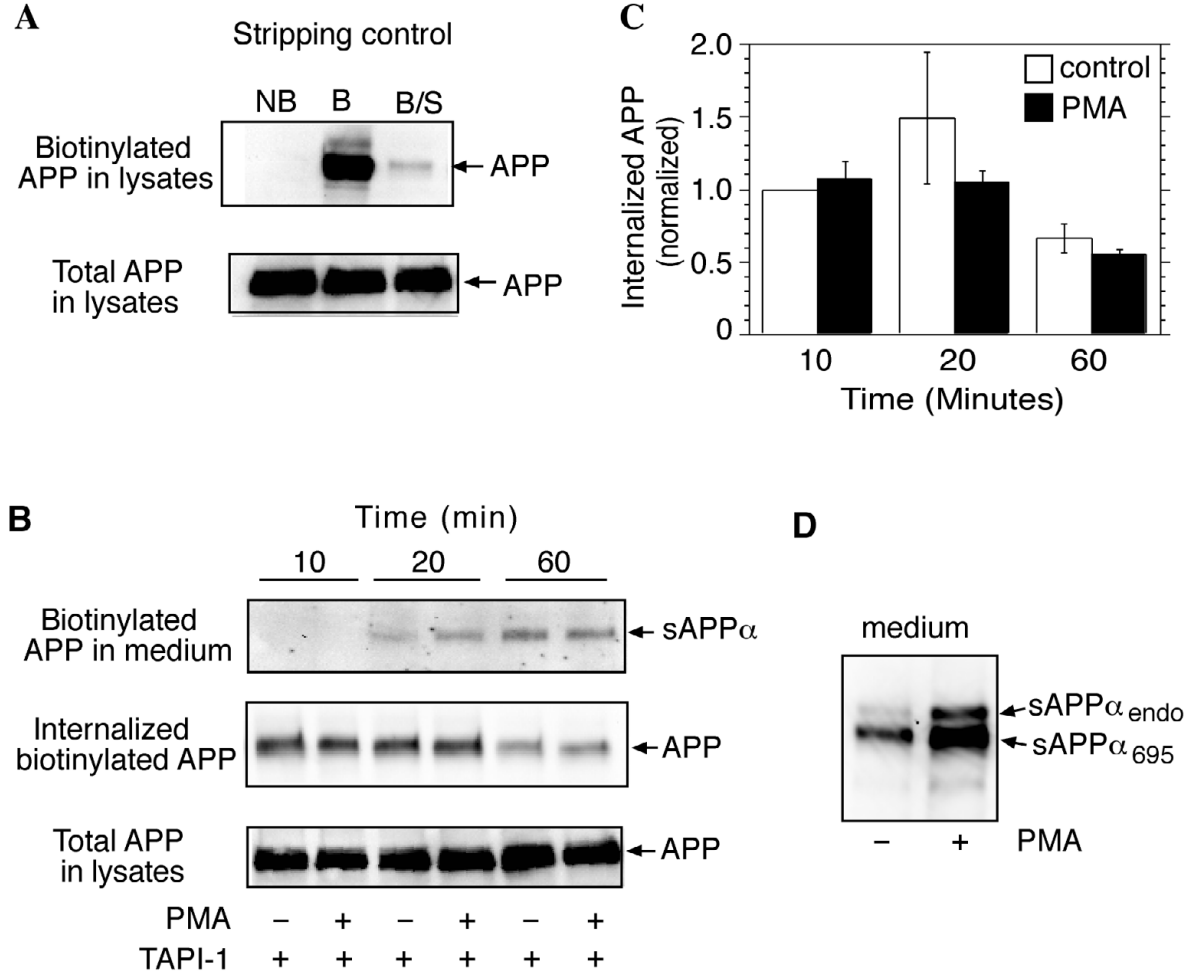
**Activation of PKC does not affect APP internalization. **HEK-695 cells were pre-treated with TAPI-1 in serum free DMEM for 1 hour prior to biotinylation. After biotinylation and quenching, cells were incubated at 37°C for various time periods in the presence of TAPI-1 and either DMSO or PMA (1 μM). The medium was collected and biotinylated sAPPα was isolated and analyzed by immunoblot using 6E10 antibodies. The cells were stripped and lysed, and biotinylated APP was isolated and assessed by immunoblot analysis with antibodies to the APP C-terminal. A. Internalization of biotinylated APP was nearly absent in cells that were biotinylated and stripped (B/S) while still on ice. NB, non-biotinylated; B, biotinylated, not stripped. B. PMA, in the presence of TAPI-1, did not affect release of biotinylated sAPPα(upper panel), or internalization of full-length biotinylated APP (middle panel). C. Bands depicting biotinylated and internalized APP were quantitated by densitometry, normalized, and expressed as means ± SEM from 3 experiments. D. In the absence of TAPI-1, PMA caused a marked increase in release of endogenous and transfected sAPPα from HEK-695 cells.

Activation of PKC by administration of phorbol esters, or via stimulation of receptors coupled to PKC, increases APP ectodomain shedding, but the mechanism remains unclear [7]. It is known that inhibiting APP endocytosis via mutation of internalization motifs, or truncation of the cytoplasmic domain, also increases ectodomain shedding [23-25], raising the possibility that physiological mechanisms that regulate shedding might act by targeting the endocytic machinery. To address this question, the effect of the PKC activator PMA on APP internalization in HEK cells stably overexpressing APP_695 _was determined using reversible surface biotinylation. Shedding was inhibited by incubating cells with the α-secretase inhibitor TAPI-1 for 1 hour prior to biotinylation. Cells were surface-biotinylated while on ice, then incubated at 37°C in DMEM containing TAPI-1 and either PMA (1 μM) or the vehicle dimethylsulfoxide (DMSO) for varying periods of time. Media and cell lysates were collected and processed as described (see "Methods"). As a control for stripping efficiency, some cultures were biotinylated and stripped while remaining on ice (Fig. 5A). Despite the presence of TAPI-1, there was a slight, but detectable, time-dependent increase of biotinylated sAPPα_695 _in the medium. This was not affected by PMA, indicating that TAPI-1 effectively blocked the signal-dependent release of APP (Fig. 5B, upper panel). Levels of internalized biotinylated APP declined over the 60 minute incubation period, consistent with the degradation of internalized APP, or the removal of the biotin label in an endocytic compartment. APP internalization was not significantly altered by the presence of PMA (Fig. 5B, middle panel, and 5C). In the absence of TAPI-1, PMA caused a marked increase in secretion of endogenous and transfected sAPPα from HEK-695 cells, as expected (Fig. 5D).

## Discussion

Cleavage of APP within the Aβ domain by α-secretases is of great physiological interest, not only because it precludes the formation of Aβ, but also because it generates a soluble N-terminal fragment, sAPPα, that exhibits neuroprotective properties [26,27]. Moreover, shedding of the ectodomain is a prerequisite for cleavage of the intracellular domain by γ-secretases; a process that liberates a C-terminal fragment with transcriptional activity [28-30]. Although the up-regulation of APP shedding by activation of PKC-dependent signaling pathways has been well-documented [7], the mechanism mediating this response is still obscure.

The present study was undertaken to determine if inhibitors of dynamin function would affect ectodomain shedding of APP. We first showed that APP internalization is dependent on the activity of dynamin, a large molecular weight GTPase that mediates both clathrin-dependent endocytosis, and internalization of caveolae, by promoting the separation of endocytic vesicles from the plasma membrane [22,31]. In confirmation of a recent study [20], we found that overexpression of a dominant negative dynamin mutant protein in HEK cells increased surface expression of full-length APP, and release of sAPPα. Thus, although cleavage of APP by α-secretases occurs largely in an intracellular compartment in many cell types (reviewed in [6]), our results suggest that inhibition of dynamin function, by preventing internalization of APP, increases its dwell-time on the cell surface, and prolongs its interaction with α-secretases at the plasma membrane. Similar elevations in APP secretion are induced by mutations of the APP cytoplasmic domain that inhibit internalization [23-25]. Consistent with the observed increase in α-secretase mediated cleavage, expression of the dynamin mutant increased cellular levels of C83, the C-terminal stub remaining after α-secretase-mediated cleavage of APP (Fig. 2B and 2C).

The increase in sAPPα release in HEK cells overexpressing dyn I K44A was associated with a reduction in the release of Aβ_1–40_, (Fig. 4), a result in keeping with reports that Aβ is generated in an endocytic compartment [24,25,32-34]. Our results are also in agreement with a study by Ehehalt et al. [35] who found that overexpression of a dyn K44A mutant protein reduced formation of the Aβ peptide in mouse neuroblastoma N2a cells. In contrast, Chyung and Selkoe reported that Aβ generation was increased in HeLa cells following induction of dyn K44A expression [20]. The increased Aβ formation observed in the latter study occurred in the absence of any alteration in the synthesis or maturation of APP, and suggested that, in HeLa cells, processing of APP by β- and γ-secretases occurs at the plasma membrane [20]. Indeed, an active γ-secretase complex was subsequently isolated from the plasma membrane of HeLa cells [36]. As a possible explanation for the reduction in Aβ observed by Ehehalt et al. [35] in cells overexpressing dyn K44A, Chyung and Selkoe pointed out that those workers measured formation of radiolabeled Aβ in cells labeled for 1 hour with [^35^S]methionine, and surmised that the mutant dynamin reduced generation of labeled Aβ by increasing the amount of unlabeled APP at the cell surface, and diluting the concentration of labeled precursor available for cleavage by β- and γ-secretases. In support of the notion that Aβ can be generated at the cell surface, Ehehalt et al [35] showed that when endocytosis was blocked by transfection with dyn K44A, the reduction in Aβ could be partially rescued by antibody cross-linking of APP and the β-secretase, β-site APP-cleaving enzyme (BACE). The decrease in total Aβ_1–40 _generation in HEK cells overexpressing dyn I K44A described in the present report might simply reflect reductions in the precursor pool due to increased cleavage of APP by α-secretase. This result is consistent with earlier studies showing that upregulation of α-secretase cleavage by PKC activation in HEK cells [37], or via mutations of the APP cytoplasmic domain in stably transfected HEK or Chinese hamster ovary (CHO) cells [23-25], is associated with decreased Aβ formation. The discrepancies among these studies might be due at least in part to cell-specific differences in the compartments where APP comes into contact with α- and β/γ-secretases, or in the relative capacities of the different secretases to cleave APP within a specific compartment.

Modulation of endocytosis might represent a mechanism for physiological regulation of APP processing by PKC-dependent signaling pathways. PKC phosphorylates dynamin, thereby activating its GTPase activity [17], and inhibiting its association with phospholipids in vitro [18]. In nerve terminals, dynamin must be dephosphorylated in order to promote retrieval of synaptic vesicles following exocytosis, and re-phosphorylation is required for the next round of endocytosis that follows a second stimulus [19]. Persistent phosphorylation of dynamin might therefore be predicted to interfere with endocytosis. Contrary to expectation, the PKC activator PMA did not affect the rate of APP internalization, as determined by reversible biotinylation in the presence of the α-secretase inhibitor TAPI-1 (Fig. 5). Thus, although PKC activation can modulate endocytosis of a variety of transmembrane proteins, either positively, in the case of β1 integrin, GABA receptors, and the dopamine transporter [38-41], or negatively, as is the case with μ-opioid receptors [42], we could not find evidence for a modulatory effect of phorbol esters on APP internalization. Others have shown that PKC activation increases APP ectodomain shedding in PC12 cells by stimulating trafficking of APP through the secretory pathway [13]. In contrast, surface expression of APP was reduced in CHO cells that were surface biotinylated following treatment with PMA and TAPI, suggesting that in these cells, PMA did not increase trafficking of APP to the plasma membrane, but possibly stimulated α-secretase-mediated cleavage within an intracellular compartment that was partially resistant to TAPI [43]. Interestingly, the motor neuron-derived trophic factor neuregulin-1, a ligand for the tyrosine kinase receptors ErbB3 and ErbB4, was found to increase the rate of internalization and degradation of APP in cultured myotubes, while decreasing release of the ectodomain [44]. This report lends credence to the hypothesis that modulation of APP internalization may represent a physiological mechanism for regulation of sAPPα release.

## Conclusion

Our results show that experimental manipulations that interfere with the function of the endocytic machinery can inhibit APP internalization, and shift APP proteolysis to a non-amyloidogenic pathway, in HEK cells. In HeLa cells, in contrast, an interfering dynamin mutant increased both α-secretase cleavage of APP and Aβ formation [20], suggesting that cell-specific differences in APP metabolism may influence the consequences of altered endocytosis. The levels of a number of proteins important for clathrin-mediated recycling of synaptic vesicles, including dynamin, and the clathrin assembly-mediating adapter proteins AP2 and AP180, are reduced in the brains of AD patients [45]. Moreover, exposure of neurons to Aβ in vitro was recently reported to reduce dynamin levels [46]. It is therefore possible that alterations in clathrin-mediated endocytosis play a role in the abnormal metabolism of APP that is characteristic of AD. Finally, given the putative role of APP as a cell surface signaling molecule in the brain [47], it is important to consider the possibility that alterations in APP endocytosis may contribute to the pathologic process by disrupting the normal signaling function of APP.

## Methods

### Materials

Antibodies and other reagents were obtained from the following sources: 6E10 antibodies to sAPPα from Signet Laboratories (Dedham, MA), antibodies to the C-terminus of APP (APP-CT) from Zymed Labs (San Francisco, CA), anti-dynamin monoclonal antibodies from BD Biosciences (San Diego, CA), goat polyclonal antibodies specific for dynamin I from Santa Cruz Biotechnology (Santa Cruz, CA), and goat anti-mouse IgG and goat anti-rabbit IgG peroxidase-conjugated secondary antibodies from BioRad (Hercules CA). Immunofluorescence-conjugated secondary antibodies including Alexa Fluor 488-conjugated goat or donkey anti-mouse IgG, and Alexa Fluor 594-conjugated rabbit anti-goat IgG, and ProLong Anti-fade mounting medium were obtained from Molecular Probes (Eugene, OR). Mini-gels and reagents for electrophoresis were obtained from BioRad (Hercules CA), and polyvinylidene difluoride (PVDF) membranes were purchased from Perkin-Elmer (Boston, MA). The metalloproteinase inhibitor, tumor necrosis factor-α protease inhibitor (TAPI-1), was obtained from Peptides International (Louisville, KY). 2-Mercaptoethanesulfonic acid sodium salt, iodoacetamide, and phorbol 12-myristate 13-acetate (PMA) were obtained from Sigma-Aldrich (St. Louis MO). Sulfo-NHS-SS-Biotin was purchased from Pierce (Rockford, IL), Other reagents and materials were acquired from Fisher Scientific (Pittsburgh PA).

### Cell culture

HEK-M3 cells (HEK cells stably transfected with M3 muscarinic receptors) and HEK-695 cells (HEK cells stably overexpressing APP_695_; a gift from Dr. Dennis Selkoe) were grown in Dulbecco's Modified Eagle Medium (DMEM)/F-12 supplemented with 10% Fetal Bovine Serum (Invitrogen Life Technologies, Carlsbad, CA) and maintained at 37°C in an atmosphere of 95% air, 5% CO_2_. HEK-M3 cells were used in some of these studies because the regulation of constitutive and receptor-coupled sAPPα release has been well characterized in this line [5,48,49].

### Transient transfections

Cells were transiently transfected with plasmids encoding APP_695 _(a gift from Dr. Carmela Abraham) and dyn I K44A (a gift from Dr. Marc Caron), or with an empty pcDNA3 vector, using Lipofectamine Plus™ reagent (Invitrogen Life Technologies, Carlsbad CA) according to the manufacturer's specifications. Experiments were carried out 48 hours later.

### Cell surface biotinylation

Confluent HEK cells were pre-incubated in serum-free DMEM for 2 hours, then washed in phosphate buffered saline (PBS), pH 7.9, supplemented with 1 mM Ca^++ ^and 2 mM Mg^++^. Surface biotinylation was carried out by incubating the cells for 30 min on ice with Sulfo-NHS-SS-Biotin (0.5 mg/ml in PBS). Culture dishes were kept on ice in the dark and gently rocked during the incubation period. The biotin reagent was quenched by treating the cells with two 15 min washes of 50 mM glycine in PBS. Cells were rinsed again with PBS and lysed in a buffer containing 50 mM Tris-HCl (pH 7.5), 150 mM NaCl, 2 mM 4-(2-aminoethyl)benzenesulfonyl fluoride, 1 μg/ml leupeptin, 1% (v/v) Nonidet P-40, 0.05% (w/v) sodium dodecyl sulfate, 0.5% (w/v) deoxycholate. Lysates were incubated overnight with streptavidin-coated agarose beads (Pierce, Rockford, IL) at 4°C in a rotary mixer to isolate biotin-labeled proteins. Isolates were size-fractionated on SDS gels, and analyzed for APP content by immunoblotting.

### Reversible biotinylation

HEK cells were pre-treated for 1 hour at 37°C in serum-free DMEM containing TAPI-1 (50 μM), then surface-biotinylated as described above. Cells were then incubated at 37° for various time periods in the presence of TAPI-1 and either PMA (1 μM) or DMSO (vehicle control). The cells were placed on ice and the remaining surface biotin was removed by applying two 20 minute washes of a stripping buffer (50 mM 2-mercaptoethanesulfonic acid (sodium salt); 150 mM NaCl; 1 mM EDTA and 0.2% BSA in 20 mM Tris, pH 8.6). The 2-mercaptoethanesulfonic acid was quenched with a buffer containing iodoacetamide (50 mM iodoacetamide, 1% BSA in PBS, pH 7.4) for 30 minutes, and cells were rinsed with PBS. To assess the efficiency of the stripping procedure, some cultures were biotinylated and then stripped while remaining on ice.

### Western blotting

The protein content of cell lysates was measured using the bicinchoninic acid reagent (Sigma, St Louis MO). Medium was collected, cleared by centrifugation, desalted, lyophilized, and resuspended in SDS-PAGE loading buffer, as previously described [5]. Lysates were centrifuged to remove insoluble material, and diluted in 2X loading buffer. Samples were normalized for protein content and size-fractionated on 7.5% or 10–20% Tris-HCl mini-gels. Proteins were transferred to PVDF membranes, which were then blocked in 5%-powdered milk in Tris-buffered saline with 0.15% Tween-20 for 2 hours, and probed overnight with primary antibodies. The next day, membranes were washed, and incubated with goat anti-mouse IgG or goat anti-rabbit IgG peroxidase-conjugated secondary antibodies and bands were detected using an enhanced chemiluminescence reagent (Western Lightning, Pierce). Membranes were imaged on a Kodak 440CF Image Station and quantitated using Kodak 1D Image Analysis software.

### Immunofluorescence microscopy

Cells were plated on nitric acid-washed coverslips coated with poly-D-lysine and placed in 30-mm tissue culture dishes. After 48 hours in growth medium, live cells were washed with PBS, and incubated on ice with 6E10 antibodies (at a dilution of 1:200 in PBS) to label surface APP. Cells were then transferred to an incubator and maintained at 37°C for various time periods. Cells were fixed in 3.0% paraformaldehyde in PBS for 10 minutes at room temperature, permeabilized in 0.1% Triton X-100, and blocked in 1% bovine serum albumin in PBS. APP was detected by incubating the cells with goat anti-mouse antibodies conjugated with Alexa Fluor 488. When double-labeling of APP and dynamin was required, cell preparations were incubated with goat anti-dynamin I primary antibodies (1:400), washed, then incubated with donkey Alexa Fluor 488-conjugated anti-mouse IgG, and rabbit Alexa Fluor 594-conjugated anti-goat IgG (1:200). After washing in PBS, cells were mounted with ProLong Anti-Fade mounting medium and left overnight to dry. Specimens were examined using a conventional fluorescence microscope equipped with appropriate band-pass filters, and images were captured with a Spot RT-KE camera (Diagnostics Instruments, Sterling Heights MI).

### Aβ measurement

HEK-695 cells were plated and transiently transfected with dyn I K44A or with empty vector, as described above, and allowed to grow for 48 hours. The growth medium was removed, and the cells were rinsed with serum-free DMEM. Fresh DMEM was then placed on the cells and they were incubated overnight. The next day, the medium was collected and a 1 ml aliquot was analyzed by enzyme-linked immunosorbent assay (ELISA) using a kit from Signet Laboratories (Dedham MA). Standards and samples were prepared and incubated in the plate overnight at 4°C. The ELISA was performed the next day according to the manufacturer's instructions.

## Authors' contributions

RMC carried out the immunoblotting, biotinylation and immunofluorescence studies, and helped to draft the manuscript. BAB participated in the transfection and immunoblotting experiments. IL-C contributed to the design and execution of the immunofluorescence studies. BES conceived of the study, participated in its design, and assisted in drafting and editing the manuscript. All authors read and approved the final manuscript.
